# ID 252 - Dupilumabe em Comparação ao Omalizumabe para Asma Grave com Fenótipo Alérgico no SUS

**DOI:** 10.5327/2237-9622.2025.v34s1.252

**Published:** 2025-11-25

**Authors:** Frederico Jose Bighetti Magro, Frederico Jose Bighetti Magro, Aline Garcia Barbosa, Greice Ballardin Miotto Buttelli, Renata Prioli, Alexandre Taminato, Sarah Franco Watanabe, Bianca Salvador, Bruna Marmett, Celina Borges Migliavaca, Gilson Dornelles, Nayê Balzan Schneider, Maicon Falavigna

**Keywords:** asma, imunoterapia, custos e análise de custo, análise de impacto orçamentário

## Abstract

**Introdução:**

A asma é caracterizada por inflamação crônica das vias aéreas, possuindo diferentes endotipos e fenótipos, destacando-se a inflamação tipo 2 com fenótipos eosinofílico e alérgico, para os quais imunobiológicos têm se mostrado eficazes. O omalizumabe (OMA) é o único agente disponível no SUS para o fenótipo alérgico, mas apresenta restrições de uso baseadas no peso e nos níveis séricos de IgE, com 16,6% dos pacientes não sendo elegíveis. O dupilumabe (DUPI) surge como alternativa para a asma grave com fenótipo alérgico.

**Método:**

Evidência avaliada por revisão sistemática seguida de metanálise de comparações indiretas. O consumo médio de OMA por paciente foi verificado pela análise das Autorizações de Procedimentos Ambulatoriais (Apac) emitidas em 2022 e 2023. O custo unitário do OMA foi avaliado por registros no Painel de Preços e no Banco de Preços em Saúde (abril de 2023 a março de 2024); calculou-se o desconto praticado frente ao Preço Máximo de Venda ao Governo (PMVG), ponderado pelo volume adquirido e ajustado para o Imposto sobre Circulação de Mercadorias e Serviços (ICMS) estadual. Para o DUPI, foi considerado o preço de incorporação para dermatite atópica (R$ 4.923,93 por caixa). Realizou-se análise de custo-minimização com horizonte de 20 anos e taxa de desconto de 5% para custos. Para a análise de impacto orçamentário (AIO), estimou-se o número de pacientes por regressão linear a partir do quantitativo em uso do OMA conforme Apac. Considerou-se inelegibilidade ao OMA em 16,6% dos pacientes, sendo estes elegíveis ao DUPI. Projeções foram feitas para cinco anos, considerando que 50% dos pacientes usarão DUPI no ano 1, atingindo 90% no ano 5.

**Resultados:**

Comparado ao OMA, o DUPI reduziu a taxa de exacerbações em 32% (RR 0,68; IC 95% 0,54 a 0,88; certeza baixa) e mostrou aumento no volume expiratório forçado no primeiro segundo (VEF1) em 0,08L (IC 95% 0,03 a 0,13; certeza baixa), além de apresentar redução não significativa de 26% no risco de descontinuação por eventos adversos (RR 0,74; IC 95% 0,28 a 1,93; certeza moderada). Identificamos 30 registros de preços para OMA 150 mg, apresentando desconto médio de 2,91% frente ao PMVG, resultando em custo ajustado de R$ 2.180,33 por frasco (ICMS 18%). Identificamos 17.292 Apac para OMA (código do Sistema de Gerenciamento da Tabela de Procedimentos, Medicamentos e Órteses, Próteses e Materiais Especiais do SUS – Sigtap: 06.04.84.001-2), referentes a 134 pacientes, em janeiro de 2022, e 1.316, em dezembro de 2023, com consumo médio mensal de 3,4 frascos por paciente. O consumo anual estimado foi de 40,85 frascos de OMA, representando um custo médio de R$ 89.192,47 por paciente. Para DUPI, o custo anual foi estimado em R$ 65.595,84 no primeiro ano e R$ 63.330,97 a partir do segundo ano. Em 20 anos, o uso de DUPI resultaria em economia de R$ 336.141,17 por paciente em relação ao OMA. Na AIO, estimamos 2.196 pacientes no ano 1 e 4.561 no ano 5, incluindo 704 inelegíveis a OMA que passaram a ser tratados com DUPI. O custo no cenário atual é de R$ 1,51 bilhão em cinco anos, passando a R$ 1,32 bilhão com a incorporação do DUPI, gerando uma economia de R$ 185,4 milhões em cinco anos. Ademais, análise de sensibilidade mostrou que, no cenário com 100% de participação de DUPI, a economia seria de aproximadamente R$ 300 milhões.

**Conclusão:**

Neste estudo, mostrou-se a integração entre diferentes metodologias e bases de dados para suportar a tomada de decisão, incluindo comparações indiretas, estimativa de custos ajustados ao ICMS estadual e análise das APACs para avaliar consumo médio e projeções de demanda na AIO. O DUPI possui custo inferior ao OMA, tornando-se uma opção custo-efetiva para o SUS e permitindo tratar pacientes inelegíveis ao OMA, sem adicionar custos ao sistema.

